# Mutational signatures in tumours induced by high and low energy radiation in *Trp53* deficient mice

**DOI:** 10.1038/s41467-019-14261-4

**Published:** 2020-01-20

**Authors:** Yun Rose Li, Kyle D. Halliwill, Cassandra J. Adams, Vivek Iyer, Laura Riva, Rashid Mamunur, Kuang-Yu Jen, Reyno del Rosario, Erik Fredlund, Gillian Hirst, Ludmil B. Alexandrov, David Adams, Allan Balmain

**Affiliations:** 1 UCSF Helen Diller Family Comprehensive Cancer Center, University of California San Francisco, San Francisco, CA 94158 USA; 2 Department of Radiation Oncology, University of California San Francisco, San Francisco, CA 94143 USA; 3 Abbvie, Redwood City, CA 94063 USA; 4 Nuffield Department of Medicine, University of Oxford, Oxford OX7DQ, UK; 5 Experimental Cancer Genetics, Wellcome Trust Sanger Institute, Hinxton, Cambridge, CB10 1HH UK; 6 Department of Pathology, University of California Davis Medical Center, Sacramento, CA USA; 7 Doublestrand Bioinformatics, 11331 Stockholm, Sweden; 8 Department of Cellular and Molecular Medicine and Department of Bioengineering, Moores Cancer Center, University of California, San Diego, La Jolla, CA 92093 USA; 9 Department of Biochemistry and Biophysics, University of California San Francisco, San Francisco, CA 94158 USA

**Keywords:** Cancer, Cancer genetics, Cancer genomics, Genomic instability, Sequencing

## Abstract

Ionising radiation (IR) is a recognised carcinogen responsible for cancer development in patients previously treated using radiotherapy, and in individuals exposed as a result of accidents at nuclear energy plants. However, the mutational signatures induced by distinct types and doses of radiation are unknown. Here, we analyse the genetic architecture of mammary tumours, lymphomas and sarcomas induced by high (^56^Fe-ions) or low (gamma) energy radiation in mice carrying *Trp53* loss of function alleles. In mammary tumours, high-energy radiation is associated with induction of focal structural variants, leading to genomic instability and *Met* amplification. Gamma-radiation is linked to large-scale structural variants and a point mutation signature associated with oxidative stress. The genomic architecture of carcinomas, sarcomas and lymphomas arising in the same animals are significantly different. Our study illustrates the complex interactions between radiation quality, germline *Trp53* deficiency and tissue/cell of origin in shaping the genomic landscape of IR-induced tumours.

## Abstract (web summary)

Mutational signatures induced by ionising radiation remain largely unexplored. Here in TP53 mutant mice, the authors characterise the genomic landscape of tumours induced by high- and low-energy radiation.

## Introduction

Carcinogens in the environment contribute to cancer development by inducing DNA damage, resulting in specific patterns of mutations that can be diagnostic for the causative carcinogenic agent. Such “mutational signatures” help us to understand and quantify the impact of potential carcinogens on human tumour DNA, and can provide information on the mechanisms by which these agents act. Mutational signatures have been detected in thousands of human cancers^1^, as well as in mouse models of chemical carcinogen-induced tumours^2,3^. In contrast, while ionizing radiation through medical exposures has been associated with increased risk of secondary malignancies^4^, we presently have no detailed knowledge of the types of signatures that are induced by this common source of DNA damage.

Environmental and therapeutic IR can exist in a number of different forms, depending on radiation quality and energy, which impact how radiation interacts with biological target molecules, such as DNA. High energy gamma rays or photons are the most commonly used form of therapeutic IR and occur naturally in the earth’s atmosphere due to radioactive decay or are secondary to the action of cosmic rays. In contrast, heavy-ion or high linear energy transfer (LET) radiation is characterised by charged, high energy particles (protons, Fe-ions) and accounts for approximately 1% of cosmic rays^5,6^. Individuals at highest risk from radiation exposures are patients being treated for cancer by localised radiotherapy, most often using fractionated high doses of gamma rays to focally targeted sites. However lower doses of radiation also have biological effects, the consequences of which have not been adequately investigated in terms of possible genomic changes.

In fact, the exact mechanisms through which radiation induces DNA damage and its impact on the genomic landscape of radiation-induced malignancies are not well-established and have been highly controversial. Although there is general support for a direct model in which IR causes single or double strand breaks by direct collision with DNA, indirect models have also been proposed by which IR interacts with other molecules (e.g., water) to increase DNA damage secondary to free radical formation, or impacts the normal tissue microenvironment to promote cytokine production and transformation through mechanisms that are presently unclear^7^. Radiation has been proposed to cause transformation due to a “bystander effect” even in cells that have not been directly traversed by radiation particles^8,9^ but the role of this mechanism in vivo has not been established. Resolution of these questions is important not only for our understanding of mechanisms of carcinogenesis after therapeutic or accidental exposure to ionizing radiation, but also for assessment of cancer risk as a result of occupational exposure, or, increasingly, as a consequence of longer term exposure to cosmic radiation during space flights^10,11^.

Characterising these mechanisms in human tumours is impeded by limited sample size, germline heterogeneity, complex environmental exposures and uncertainty in the dose, duration, frequency and quality of radiation exposure. Thus, the use of appropriate model systems that properly recapitulate the effects of RT in cells and animal models is pivotal. Indeed, high and low LET studies have been carried out on cells in vitro and in lower organisms, and the types of changes seen depend on radiation quality (reviewed in Durante & Cucinotta)^12^. Specifically, high LET radiation is associated with intense focal damage caused by the traversal of heavy ions through the nucleus^13–15^. In contrast, gamma radiation causes more indirect DNA damage, diffuse patterns of H2AX foci, and increased oxidative stress^16^.

*Trp53*, a gene also known as the guardian of the genome^17^, is critical in affecting the cellular outcome following DNA damage^18,19^. Human patients with germline *TP53* mutations are highly tumour-prone^20,21^, and genomic studies have linked germline *TP53* deficiency to development of genomically unstable tumours with a high incidence of chromothripsis^22–25^. To develop a deeper understanding of the effects of radiation on tumour DNA integrity, we use mouse strains with partial germline deficiency in *Trp53* function, i.e., mice hemizygous for *Trp53* (*Trp53*^(*+/−*)^), or homozygous for the Delta P *Trp53* allele (*Trp53*^(*DP/DP*)^); hereafter *Trp53ΔP*). The *Trp53*^(*+/−*)^ strain is highly susceptible to both spontaneous and radiation-induced (4 Gy gamma) lymphoma development, while the *Trp53ΔP* strain develops a wider range of tumours after exposure to the same radiation dose^26–28^. The *Trp53ΔP* mouse carries a deletion in the N-terminal proline-rich domain (PRD) of TP53, a region that is important in the regulation of human TP53 activity and stability. A common germline polymorphism at codon 72 in this region has been associated with altered cancer risk in humans^29,30^.

In this study, we breed both strains of mice on to the same genetic background (FVB/N), and expose the animals to a relatively low dose (50 cGy) of gamma (low LET) or Fe-ion (high LET) radiation to explore the effects on susceptibility to tumour development in different tissues. In particular, the 50 cGy dose of Fe-ions is studied as it has been proposed by NASA and other previous studies as a realistic dose to which astronauts may be exposed during exploratory space missions^31–33^. We demonstrate that this low radiation dose results in the development of a wide range of malignancies in the context of germline *Trp53* deficiency, but particularly in a high incidence of mammary carcinomas and angiosarcomas, which are also common radiation-associated malignancies in humans. Whole-genome sequencing of mammary tumours and whole-exome sequencing of a wider spectrum of IR-induced tumours reveals complex point mutational signatures and patterns of structural rearrangements in tumour genomes that are associated with radiation quality, as well as germline *Trp53* status and tumour histology.

## Results

### Low dose radiation-induced tumours in Trp53-deficient mice

**Fig. 1:**
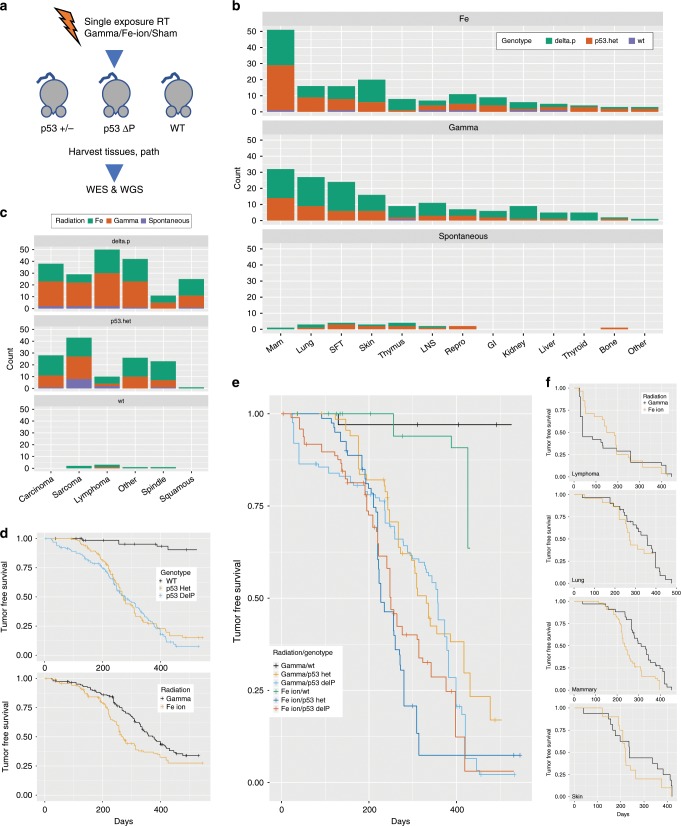
Tumour histology and survival in observed RT-induced malignancies. **a** Study schema showing that animals of different germline *Trp53* status (Trp53+/−, *ΔP* (*Trp53ΔP*), and WT) receiving radiation of different qualities (Gamma, Fe-Ion and Sham) developed a range of different tumour types that were harvested and studied for pathology and selectively sequenced by whole-exome (WES) or whole-genome (WGS) platforms. **b** Tissue distribution of different tumour types following whole body radiation exposure, as a function of radiation quality and germline *Trp53* status. Independent tumours from animals (with clearly independent primaries by histology) were separately counted. SFT soft tissues, LNS Lymph nodes, Repro, reproductive organs, GI gastrointestinal tract. *N* = 479. **c** Incidence of different pathologic tumour types by radiation quality and germline *Trp53* status. Most adenocarcinomas and sarcomas were in the mammary gland, and most squamous carcinomas in the skin. *N* = 479. **d** Tumour-free survival (TFS) of animals by germline genotype (top) and radiation quality (bottom). An event was defined death due to tumour confirmed by pathology and dissection and/or visible tumour requiring euthanasia. *N* = 238. **e** Interaction of genotype and radiation quality on TFS. Wildtype animals with exposure to Fe-Ion have a substantial risk of malignancy as compared to animals with gamma exposure. *N* = 238. **f** TFS related to different tumour disease sites. Survival was reduced in animals developing skin, mammary or lung tumours after exposure to Fe ions compared to gamma radiation, but this was reversed for lymphomas, which showed earlier onset and were preferentially induced by gamma radiation exposure. *N* = 238.

We previously showed that *Trp53ΔP* mice in a mixed 129/C57BL6 background were highly sensitive to development of several tumour types after a single exposure to whole body gamma radiation, but had a low spontaneous rate of tumour development^26^. We exposed 238 FVB/N mice that were *Trp53* WT, hemizygous (*Trp53*^*+/−*^) or homozygous for the Delta P *Trp53* allele (*Trp53ΔP*), to a single dose of either ^56^Fe-ion (600 MeV/amu) or gamma radiation. Most animals were exposed to a dose of 50 cGy of either radiation type, but a few received lower or higher doses (Supplementary Data 1 and Suppementary Table 1). Animals were followed for >400 days to monitor tumour development; a subset of sham irradiated controls was also included (Fig. 1a). A total of 160 mice developed at least one of several histological types of tumour after radiation exposure, most commonly in the mammary glands, skin, lung and lymphoid tissue (Fig. 1b, c). As expected, wild type mice were generally resistant to development of tumours induced by radiation (Fig. 1d), but a few animals were sacrificed early, predominantly those that developed tumours after exposure to ^56^Fe-ions (Fig. 1e). Overall, *Trp53ΔP* animals were much more likely to develop carcinomas in the skin, mammary gland, and other tissues, versus other histologic subtypes, (*P* *=* 3.63e−05, chi-sq test; Fig. 1b, c).

The most common site of tumour development was the mammary gland, regardless of radiation quality or *Trp53* germline status (Fig. 1b). There were a number of histological subtypes, including adenocarcinomas (ADC), squamous cell carcinomas (SCC), spindle cell carcinomas (SPN), and sarcomas (SAR), including angiosarcomas (Fig. 1c). Notably, in humans, sarcomas and angiosarcomas appear to show a disproportionally higher incidence in patients with a history of prior radiation therapy to the chest or thorax^34^. Compared to other models of radiation-induced tumours in *Trp53*-deficient mice (129/Sv strain, 4 Gy gamma ray exposure) where the predominant tumour types were lymphomas and sarcomas^27,35^, the present model (i.e., FVB/50 cGy) more closely mimics the spectrum of radiation-induced tumours observed in humans.

Radiation quality had a significant impact on tumour latency and disease-free survival (DFS), as animals exposed to Fe-ion radiation survived on average 287 days, as compared to gamma radiation (358 days) (univariate Cox-Proportional Hazard analysis, HR = 2.36, *P* < 1.94e−06; Fig. 1d–f). No obvious effect on survival was attributable to mouse strain genotype (*Trp53*^(*+/−*)^ or *Trp53ΔP*, Fig. 1d). On analysis by disease site, mammary, skin, and lung tumours all had a shorter latency following exposure to ^56^Fe radiation compared to gamma. In contrast, a different pattern was seen for lymphomas, which developed earlier after exposure to gamma radiation (Fig. 1f, lower right panel).

### Mutational signatures in radiation-induced tumours

We performed whole-genome sequencing (WGS) of a focused cohort of 21 mammary tumours from animals of *Trp53*+/− or *Trp53ΔP* backgrounds exposed to 50 cGy Fe-ion or gamma radiation (Supplementary Table 1). The overall frequency of point mutations or single nucleotide variants (SNVs) was low (median = 1.16, mean = 2.009; range 0.62–14.6 SNVs/Mb), considerably less than observed in chemically-induced mouse tumours of the same genetic background^2,3^. The rate did not differ significantly according to tumour type or radiation quality (*P* = 0.52 and *P* = 0.92, respectively, Fisher’s Exact test; Supplementary Fig. 1A). The dearth of point mutations in these samples, most of which mapped to non-coding regions of the genome (Supplementary Fig. 1C and D), is consistent with that reported previously in radiation-associated human tumours^4^.

**Fig. 2:**
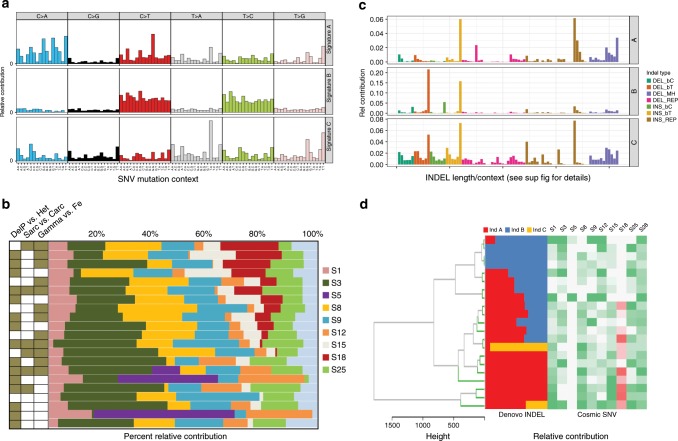
SNV and INDEL signatures in RT-induced mammary tumours. **a** De novo SNV signature extraction using NMF identified three distinct point mutation signatures, referenced to here and in the text as “A”, “B” and “C”. *N* = 21. **b** Deconvolution of the mutation signatures into known Cosmic signatures found in human tumours. Samples are denoted by radiation quality, tumour pathology and *Trp53* genotype. DelP (Brown) = P53 Delta Proline (*Trp53ΔP*); Het = P53 Heterozygous (White), Sarcoma (Brown), Carcinoma (White), Gamma (Brown) and Fe (White). The red blocks represent the contribution of the signature 18 “ROS” to total mutations in each sample, and these are relatively enriched in tumours induced by gamma radiation compared to ^56^Fe ions (*P* < 0.032). Pale blue denotes contribution from variants not explained by known Cosmic signatures. *N* = 21. **c** Relative contribution of each insertion/deletion mutation type in the three INDEL signatures identified in the mammary tumour samples. See Supplementary Fig. 2 for a breakdown of all categories of INDEL types. “Del_B” = 1 bp deletion, “Ins_B” = 1 bp insertion, where “C” or “T” describes whether cytosine or thymidine was deleted at that position, “MH” = microhomology domain and “REP” = >1 bp deletion or insertion at repeats. *N* = 21. **d** Relationship between INDEL signatures and SNV signatures. Hierarchical clustering of both INDEL signatures and SNV signatures demonstrates an enrichment of de novo INDEL signature A (left) and SNV signature 18 (ROS, right) in samples induced by exposure to gamma radiation compared to those induced by ^56^Fe ions (*P* < 0.032). *N* = 21.

As SNV mutational signatures can provide genomic evidence for the aetiology of tumour initiation and progression, we next evaluated whether tumours that result from exposures to different radiation qualities show distinct point mutation patterns^1,36,37^. We hypothesised that a signature of reactive oxygen species (ROS)-related DNA damage may be observed as an indirect consequence of IR exposure. IR is known to generate free radicals^38^, which can in turn damage DNA by causing the formation of 8-Oxo-guanine adducts. We addressed this question by two different approaches: searching for de novo mutational signatures using Nonnegative Matrix Factorisation (NMF), and analysis of cosine similarity to existing trinucleotide mutational signatures in a wide range of human cancers^37^. When we performed de novo mutational signature extraction using WGS-derived SNVs, we observed 3 independent signatures (Fig. 2a). Signature SNV A was enriched for G > T (C > A) substitutions which can be generated by misrepair of 8-oxo-guanine adducts^38^. This signature is consistent with the previously described human COSMIC signature 18, which was previously reported to be associated with oxidative DNA damage due to ROS^39,40^. Signature SNV B resembles the pattern of human signature 5, which is one of the clock signatures and is comprised of C > T and T > C transitions^37^, while signature SNV C had no obvious similarity with other Cosmic signatures.

Signature deconvolution using the established human COSMIC mutational signatures^36,37^, revealed a strong cosine similarity with several human signatures, including signature 18, clock signature 5, and signature 3, which is found in most mouse mammary carcinomas and is associated with defects in homologous recombination (Fig. 2b). This process is critical for the repair of double-stranded breaks induced by RT^37^. Signature 18 (red, Fig. 2b) was variable across samples, but was preferentially enriched in tumours induced by gamma radiation, compared to those induced by ^56^Fe-ions (*p* < 0.032, Fisher’s Exact test). While these data are based on a relatively small sample size (12 gamma-induced and 9 heavy ion-induced tumours), the results are consistent with the hypothesis that 50 cGy gamma radiation results in higher levels of ROS than 50 cGy high LET radiation.

### Indel signatures in radiation-induced tumours

We performed de novo INDEL mutational signature extraction from the 21 WGS samples, identifying three unique *INDEL* signatures: *A, B* and *C* (Fig. 2c and Supplementary Fig. 2)^1^. Signature *INDEL-A* was enriched for microhomology domain-associated deletions and repeat-mediated insertions, while signature *INDEL*-*B* was enriched in single base simple Thymidine insertions and deletions. Both signatures were observed in the majority of samples without clear association with *Trp53* genotype. Signature *INDEL-C* was identified in only 2 samples and was not strongly enriched for any particular INDEL class.

Unsupervised hierarchical clustering of both INDEL and SNV signatures (Fig. 2d) revealed some significant correlations between specific combinations of INDELs and SNVs. The high ROS SNV signature (human signature 18) that was enriched in samples induced by exposure to gamma radiation (Fig. 2b) showed a significant correlation with signature *INDEL* A (*P* = 0.017, Fisher’s Exact test) (Fig. 2d). Interestingly, Sharma et al. have proposed that ROS-induced damage can cause oxidatively induced clustered DNA lesions that lead to double strand breaks and deletions caused by error-prone repair by the non-homologous end-joining (NHEJ) pathway^41^. It is therefore possible that the co-occurrence of SNV signature 18 and signature *INDEL* A is related to relatively higher levels of ROS induced by gamma rays, but further detailed studies would be required to confirm this association.

### Structural rearrangements in radiation-induced tumour genomes

**Fig. 3:**
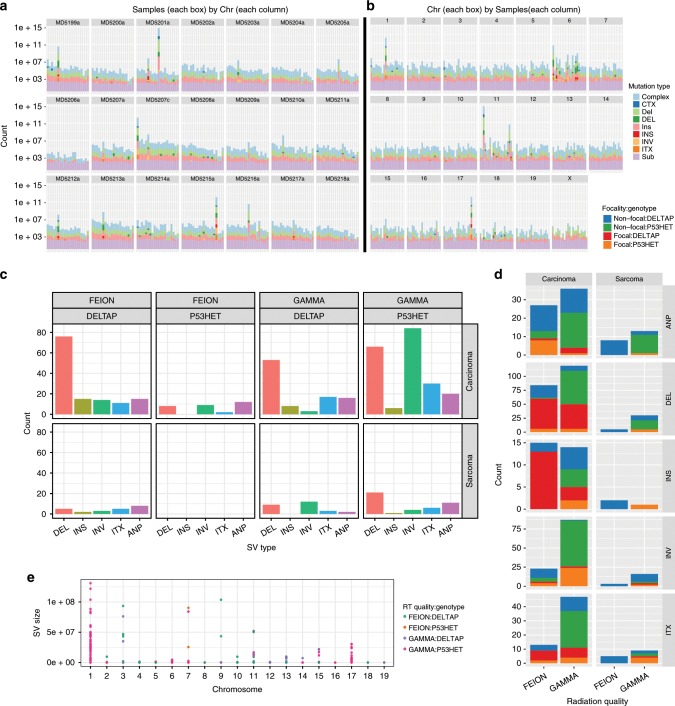
The mutation landscape of SNVs and SVs across radiation-induced malignancies. **a** Distribution of mutations identified in mammary tumours by individual samples. Each column is one chromosome (*x*-axis). Total count of mutations is on the *y*-axis. Mutations are coloured by type. Sub: Substitutions (SNVs), ITX: Intrachromosomal translocation, INV: Inversion rearrangements, INS: insertion rearrangements, DEL: deletion rearrangements, CTX: Complex or inter-chromosomal translocations, Complex: Complex insertions. Number of base pairs impacted by each mutation type is plotted on the *y*-axis in logarithmic scale. **b** Distribution of mutations (as in **a**) in each WGS mammary tumour sample sorted by chromosome. Each column corresponds to one unique sample and each facet box encompasses one chromosome. Large scale variants were found mainly in chromosomes 1, 6, 11 and 17. **c** Frequency (*x*-axis) of structural variants observed in mammary tumours based on radiation quality, *Trp53* genotype and tumour histology. ITX: intrachromosomal translocation, INV: inversion rearrangements, INS: insertion rearrangements, DEL: deletion rearrangements, ANP: aneuploidy. Carcinomas have more SVs than sarcomas from the same cohort of mice, and large inversions are enriched in tumours from *Trp53*+/− mice exposed to gamma radiation. **d** Incidence of focal and non-focal genomic events in mammary tumours based on radiation quality, germline *Trp53* genotype and tumour histology. A higher rate of focal mutations (red and orange blocks) is observed in Fe-Ion associated carcinomas. Large non-focal structural variants (green and blue blocks) were more prevalent in samples induced by gamma radiation. Mammary sarcomas have significantly fewer structural variants than mammary carcinomas from the same cohorts of mice, independent of radiation quality and *Trp53* genotype. **e** Distribution of structural variants identified in the same samples as in **c** by size across the mouse genome. Size (*y*-axis) is shown by log scale. Chromosome number is shown along the bottom. Most SVs are small and less than 1 MB, as expected.

In contrast to the low SNV rate, genomes from radiation-induced tumours bore frequent structural rearrangements. Figure 3 shows the patterns of genomic alterations in mammary tumours arranged by sample ID (Fig. 3a) or by chromosome (Fig. 3b) based on whole-genome sequencing. We identified a mean of 27 rearrangements per tumour. These large structural variants (SVs) were enriched in deletions (mean *n* = 11.3/sample) as opposed to insertions (1.52) or inversions (6.1). A total of 74 intrachromosomal (ITX) and 66 interchromosomal complex translocations (CTX) were observed. The distributions of mutations across samples and chromosomes were non-homogeneous. SVs as well as smaller INDELs were clustered in some tumours (Fig. 3a) and also were located predominantly in a few chromosomes including chromosomes 1, 6, 11 and 17 (Fig. 3b).

Carcinomas were significantly more enriched in large scale SVs than sarcomas (*P* < 0.01, Fisher’s Exact test; Fig. 3c top vs lower panels). Furthermore, inversion rearrangements were more common in carcinomas from *Trp53*+/− than from *Trp53ΔP* mice, and in particular carcinomas in mice exposed to gamma radiation carried 68.2% of all inversions (*P* < 1 × 10^*−*4^, Fisher’s Exact test; Fig. 3c (top right panel) and 3D). Prior work has shown that distinct mutational processes can impact the observed frequency of focal versus non-focal rearrangements^42^. We classified structural variants into focal and non-focal SV types and examined the differences in the distribution of focal SVs (including mixed chromosomes containing complex regions of copy number gains and losses) as opposed to non-focal rearrangements (including whole chromosome aneuploidy (see Methods section), as a function of germline *Trp53* genotype, histological subtype and radiation quality (Fig. 3d). In addition to showing that SVs are rare in sarcomas, this analysis showed that ^56^Fe-ion exposure was more likely to result in focal deletions and insertions, whereas non-focal SVs or whole chromosome aneuploidy were enriched in tumours induced by gamma radiation or from Trp53+/− mice (Fig. 3d). Most SVs were small (<100 Kb) although clusters of larger SVs were observed in several chromosomes (Fig. 3e). We conclude that the type of radiation to which the mice are exposed has differential effects on the nature of the resulting genomic alterations, with heavy ion, high LET radiation linked to more focal changes, particularly in *Trp53ΔP* mice, and low LET gamma radiation associated with larger scale chromosome alterations, particularly in *Trp53* heterozygous mice. The data therefore demonstrate a strong interaction between germline Trp53 status and radiation quality in determining patterns of genomic instability.

### Gene copy number changes in radiation-induced tumours

Analysis of gene copy number variation (CNVs) in the WGS data revealed striking patterns of gains or losses across many different chromosomes (Supplementary Fig. 3). Copy number gains were seen frequently on chromosomes 5, 6 and 15, while losses predominated on chromosomes 4, 9, 12 and 18. Interestingly, some individual tumours showed patterns dominated mainly by CNV gains (samples 2–5 on Supplementary Fig. 3 from top), losses (samples MD5208B and MD5202A), or scrambled chromosomes (sample M5215 A) in a manner reminiscent of previous analysis of CNVs in radiation-induced lymphomas using Comparative Genomic Hybridisation (CGH)^35^. Some chromosome regions showed striking focal CNV gains, for example on proximal chromosome 6.

**Fig. 4:**
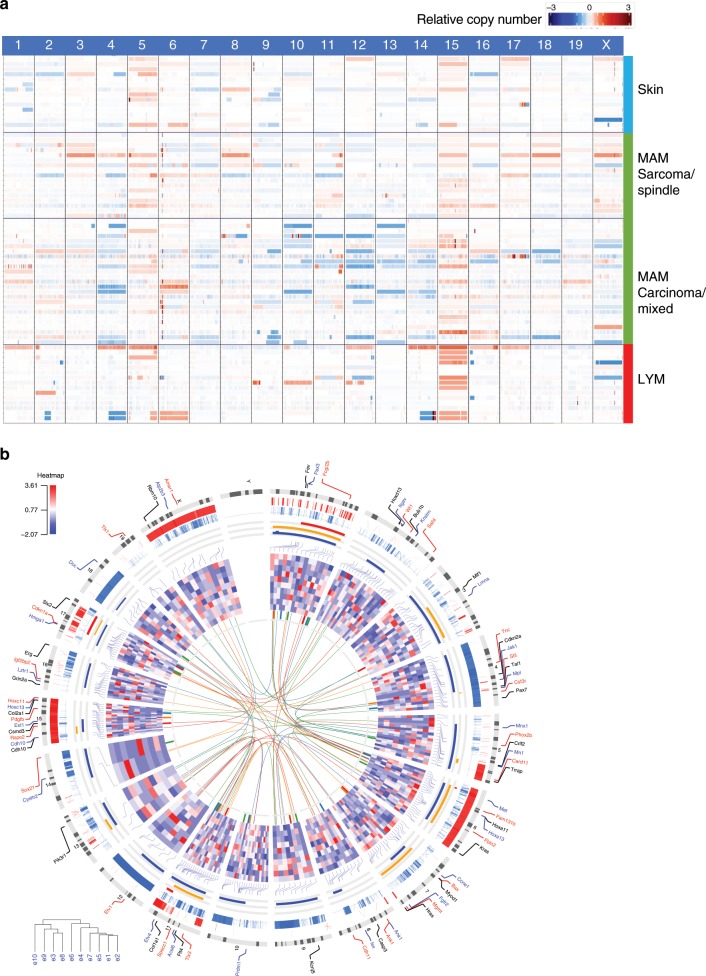
The landscape of CNVs illustrate both focal and whole-chromosome events. **a** CNVs across all sequenced (whole exome and whole genome) samples. Tumour samples are sorted by tissue site (large blocks separated by black lines) and subsequently by pathology, radiation quality and genotype status of *Trp53*. Relative copy number states across each chromosome are indicated by a corresponding key as shown at the lower right. Some chromosomes show predilection for whole-chromosomal amplification (e.g. Chromosome 5 and 15, the latter particularly in lymphomas) while other regions show more deletions (Chromosome 4) or focal amplifications (e.g., proximal Chromosome 6 corresponding to *Met* in mammary samples). **b** Circos plot illustrating relationships between amplification status, gene expression, translocations, and candidate driver genes across tumours induced by radiation exposure. From outside to inside: 1. Driver genes. 2. Cytoband map of the mouse chromosome. 3. Focal CNVs gains. 4. Focal CNVs losses. 5. Aneuploidy gains. 6. Mixed chromosome changes. 7. Aneuploidy losses. 8. Expression of cancer driver genes in tumour samples versus normal samples. 9. Intrachromosomal and interchromosomal translocations identified in samples that were subjected to whole-genome sequencing. Y-chromosome was not included in the analysis.

To validate these observations, we carried out whole exome sequencing (WES) of 56 additional radiation-induced tumours comprising a wider range of histological types (Supplementary Data 2). Combining both WGS and WES datasets, we confirmed the gains of proximal chromosome 6 in mammary tumours (Fig. 4). In contrast to these focal changes, lymphomas showed characteristic whole chromosome 15 gains, regardless of germline *Trp53* genotype or radiation quality. Chromosome 15 aneuploidy has previously been found to be a common genetic event in gamma radiation-induced lymphomas, and is thought to be driven by cMyc^35,43^. These data demonstrate the tissue specificity of genomic changes in tumours of distinct tissue and cellular origins, even in the same individual animal and after exposure to the same carcinogenic stimulus. While CNV analysis (Fig. 4) showed numerous examples of whole chromosome aneuploidy, defined as a contiguous region of gene copy number gains or losses (see Methods section), other samples had chromosomes with a mixed pattern comprising both gains and losses. The latter phenomenon was significantly correlated with radiation quality as 85% of mixed chromosomes were associated with Fe-ion exposure (*P* < 0.03). This is also consistent with the data above showing that ^56^Fe-ion exposure is associated with increased focal deletions and insertions, supporting a model in which ^56^Fe-ion radiation causes direct focal damage in DNA, leading to double strand breaks (DSB). There was also an association with tumour type, as mammary tumours were strongly deletion biased (52%) as compared to lymphoma (21%) or skin tumours (15%) (*P* < 2.9 × 10^*−*3^, chi-sq test).

Aside from chromosome 15 gains, the vast majority of aneuploid chromosomes were observed in mammary tumours. For example, loss of chromosome 4 was observed in 14 samples, 11 of which were mammary tumours. Chromosome 4 contains *Cdkn2a* encoding the *Ink4/Arf* locus strongly implicated in cancer pathogenesis with well-studied functional links to *Trp53*. Focal loss of *Cdkn2a* is a common event in chemically induced mouse tumours^3^, but such focal events were rarely detected in radiation-induced tumours. This may suggest that other genes on chromosome 4 are also driving the observed losses.

### Met is a common target of amplification in heavy ion-induced mammary carcinomas

**Fig. 5:**
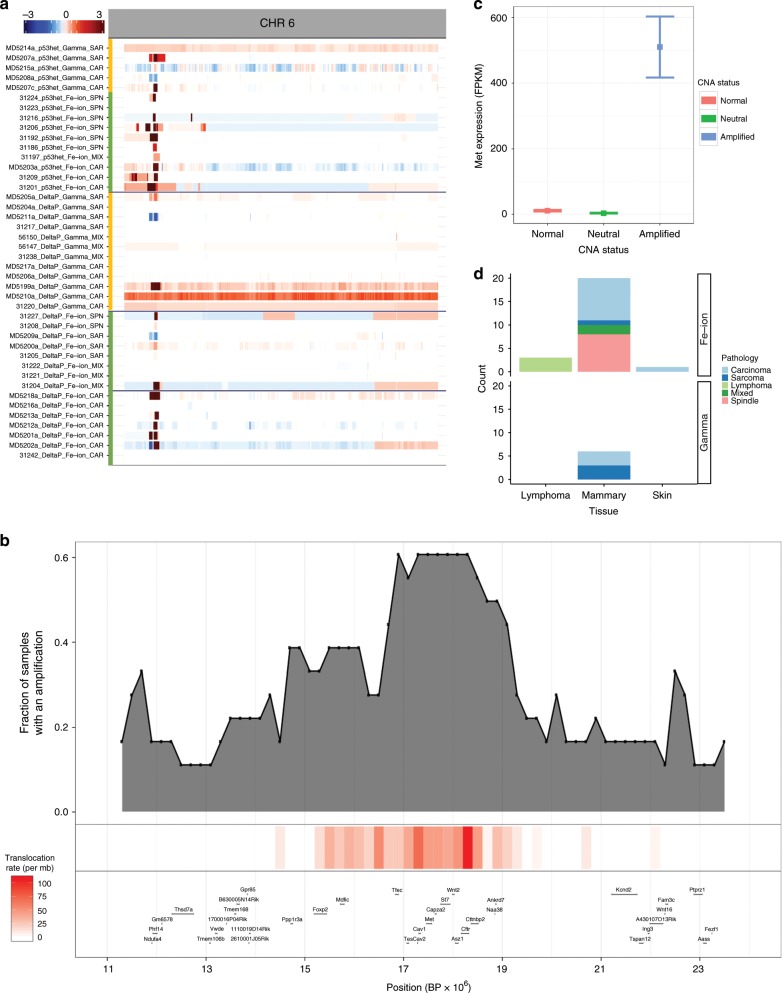
Focal amplification of *Met* is preferentially induced by ^56^Fe-ion radiation. **a** Mammary tumours bear high frequency of focal amplifications on chr 6 (at the *Met* locus) predominantly in Fe-ion associated tumours. *N* = 42. **b** Focal analysis of the *Met* locus to illustrate that the same region with focal *Met* amplifications are co-enriched for structural variants. The highest frequency of structural variants (red vertical line) does not coincide exactly with the locus with the increased copy number of *Met*, suggesting that other selection mechanisms led to the high *Met* copy number. Locations of genes in the amplified region are shown in the lower panel. *N* = 21 (translocations from WGS data). *N* = 10 (expression from RNAseq data). **c** Higher expression of *Met* by whole transcriptome RNA-seq is strongly associated with copy number status. RNA from a total of 10 mammary tumours was sequenced. Expression is shown for tumours with or without *Met* amplification, as well as matched normal samples. Error bars = 95% confidence intervals. **d** Association between *Met* amplification and tumour site of origin (mammary, skin, lymphoma), histology and radiation quality.

The proximal region of chromosome 6 encompasses the *Met* oncogene, which was amplified in a total of 23 mammary tumour samples, 19 of which were induced by ^56^Fe-ions and only 4 by gamma radiation (Fig. 5a, d). These data suggested that the focal genomic events preferentially induced by heavy ions select for mammary tumours driven by *Met* amplification. RNASeq analysis of a subset of mammary tumours confirmed that amplification of the *Met* locus was indeed associated with a large increase (>200 fold) in *Met* expression (Fig. 5c). A functional contribution from other genes in the amplified region which were also over-expressed, although not to the same level (Supplementary Fig. 4A) is however also possible. The rearrangement hotspot on chromosome six overlapped the significantly amplified region chromosome 6 cytoband A2, however the peak region of structural rearrangements did not exactly overlap with the *Met* gene (Fig. 5b). This is compatible with a model by which complex structural SVs can lead to progressive rearrangements and selection for increased copy number and expression of a strong cancer driver such as the *Met* gene^24^.

### Germline Trp53-dependent patterns of structural variants

**Fig. 6:**
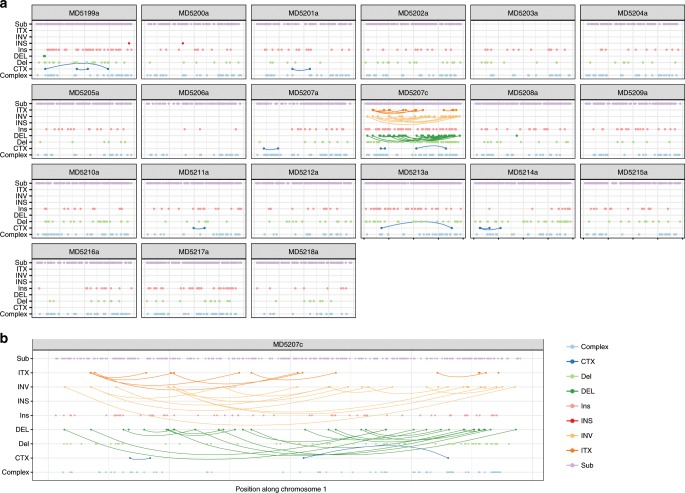
Chromothripsis in radiation-induced tumours. **a** Mapping of different somatic mutation types across chromosome 1 in all of the whole genome sequenced mammary tumours. Sub: Substitutions, ITX: Intrachromosomal translocation, INV: Inversion rearrangements, INS: Insertion rearrangements, DEL: Deletion rearrangements, CTX: Interchromosomal translocation, Ins: Small Insertions, Del: Small deletions, Complex: Complex INDELS. **b** Sample MD5207c has a strikingly large number of mutations of different types spread widely across the length of the chromosome, indicative of whole chromosome chromothripsis.

Chromothripsis, or chromosome-shattering, is a phenomenon by which numerous clustered chromosomal rearrangements occur in localised, confined genomic regions, first characterised in human tumours and some congenital diseases. This process has been attributed to a single catastrophic genetic event that results in extensive DNA damage in the context of *Trp53* dysfunction^24,25,44–46^. Chromothripsis is particularly associated with tumour development in patients with Li-Fraumeni syndrome as they bear germline *TP53* mutations^22^, but may also be more generally associated with partial insufficiency in *TP53* or other genes linked to DNA damage responses such as *ATM*^22,47^. While SVs were the predominant lesions observed in mouse radiation-associated tumours, we also saw examples of whole or partial chromosome chromothripsis. One mammary tumour (MD5207c) showed strong evidence for whole chromosome chromothripsis, with clusters of SVs across chromosome 1 (Fig. 6a, b). This mammary carcinoma was from a gamma-exposed *Trp53+/−* mouse, and showed many of the hallmarks of chromothripsis including chromosome wide translocations, deletions and inversions, with several showing overlapping breakpoints consistent with a simultaneous rather than sequential event^24^.

**Fig. 7:**
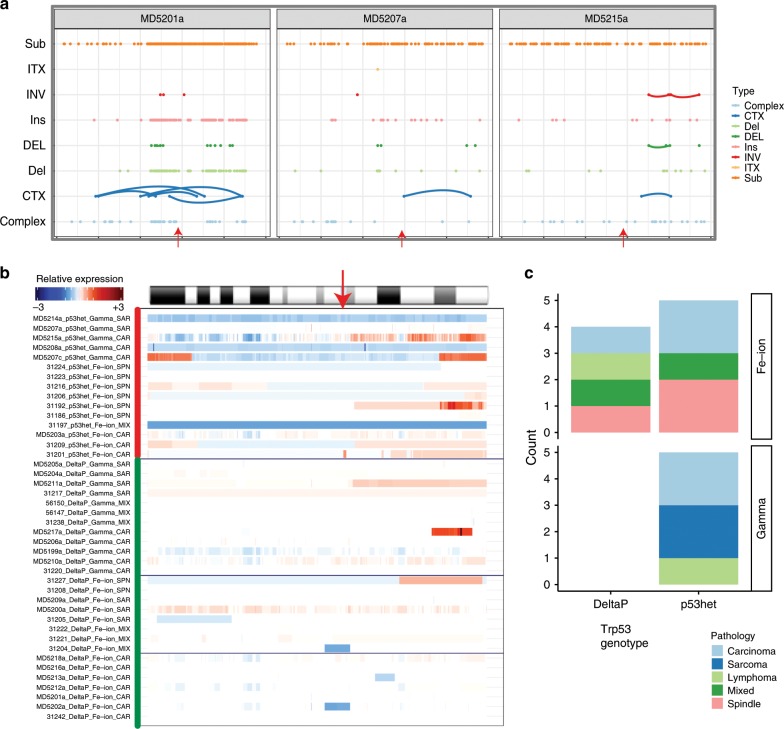
Interaction between *Trp53* genotype and RT quality in complex structural variants. **a** Three samples showing representative enrichment of SVs on chromosome 11 near the *Trp53* locus, some with evidence of co-localisation of breakpoints and/or variants. Arrows denote the location of the *Trp53* gene along chromosome 11. **b** Tumours from *Trp53*+/− mice show common non-focal deletions or whole-chromosomal aneuploidy in chr 11 (top panel). These large scale copy number alterations are less common in tumours from the *Trp53ΔP* mice (lower panels). Arrow denotes the location of *Trp53*. **c** Effect of *Trp53* germline genotype on patterns of somatic alterations at the *Trp53* gene in mammary tumours. *Trp53* deletions are frequently observed in animals of *Trp53* heterozygous background exposed to either Fe ions or gamma radiation. In *Trp53ΔP* mice, localised deletions at the *Trp53* locus were only seen in tumours induced by ^56^Fe ions.

Some carcinomas showed complex deletions on chromosome 11 overlapping *Trp53* (Fig. 7a). In total, at least five carcinomas showed focal clustered regions of SVs on chromosome 11, three of which had variants clearly disruptive of the *Trp53* locus. The two other samples demonstrated focal clustering of SVs on chromosome 11 which mapped close to but distal from *Trp53* (Supplementary Fig. 5). Notably, CNV analysis of chromosome 11 identified distinct patterns of changes in tumours from Trp53+/− and *Trp53ΔP* mice (Fig. 7b, top panel vs lower panels). Tumours from *Trp53*+/− mice showed a higher frequency of large scale or whole chromosome loss of chromosome 11, regardless of radiation quality, as a common mechanism for ensuring complete loss of *Trp53* function (Fig. 7b). In contrast, almost all changes of copy number at the *Trp53* gene in tumours from *Trp53ΔP* mice were seen in ^56^Fe-ion radiation-exposed animals (Fig. 7c), suggesting that focal DNA damage of the type induced by heavy ions is more likely to result in loss of *Trp53* in mice with this mutant allele, which is known to retain some residual *Trp53* function^28^.

### Common driver events in radiation-induced tumours

Several other potential driver genes in radiation-induced tumours were identified by a combination of CNV analysis, exome sequencing to identify point mutations, and gene expression (Supplementary Data 3). One amplicon seen in several samples was on proximal chromosome 9 in a region harbouring a family of genes encoding matrix metalloproteinases (Mmps). MMPs can regulate the tumour microenvironment, and their expression and activation is increased in tumour tissue compared to normal^48^. Specific *Mmp* genes, including *Mmp 13, 12, 10* and *3*, were highly expressed in tumours carrying the amplified region (Supplementary Fig. 4B). However alternative candidates *Birc2, Birc3* and *Yap1*, also localised in the amplified region, showed some level of over-expression and may also be functionally significant.

We also observed recurrent gene-level events in *Notch1*, a gene known to play an important role in radiation-induced lymphoma development^49^. In total, five lymphoma samples had distinct *Notch1* mutations. In two lymphomas, we found frameshift indels in the PEST domain, resulting in activation of Notch signalling, as well as multiple nonsynonymous SNVs in *Notch1* (Supplementary Data 3 and 4). Unlike the indels, these were not limited to the PEST domain and their functional implications are unclear. In two gamma radiation-induced lymphomas we found focal amplification of *Notch1*, one of which also carried a point mutation, demonstrating the multiplicity of mechanisms leading to activation of *Notch1* in radiation-induced lymphomas. Finally, significant SNVs were found in a range of known driver genes including *Kras, Apc, Pten* and hotspot sites in *Trp53*, the latter examples occurring in tumours from the *Trp53ΔP* mice (Supplementary Fig. 6 and Supplementary Data 3). The spectrum of genomic alterations, both focal and large-scale, found in these radiation-induced tumour samples is complex and reflects the heterogeneity of genomic consequences resulting from radiation exposure. The changes found over all samples is summarised in the Circos plot in Fig. 4b.

## Discussion

We have explored the impact of both high and low energy radiation on tumour development in mice carrying two distinct types of germline mutation in the *Trp53* gene. Our data demonstrate that tumours induced by both high and low LET radiation exhibit the common features of low SNV burden, significant genomic instability as shown by detection of large-scale structural variants, and evidence of whole or partial chromosomal chromothripsis. However, radiation quality had a significant impact, together with germline *Trp53* status, on the number and types of tumours that developed, the overall survival of affected mice, and the classes of genomic aberrations found by whole genome and exome sequencing. The low SNV burden and high indel/SNV ratio in mouse mammary tumours mirrors results obtained by WGS analysis of a set of 12 human tumours that were associated with prior exposure to radiotherapy^4^, but contrasts with the results obtained by analysis of tumours induced in NF1-deficient mice by high dose fractionated ionising radiation^50,51^. In the latter studies based on exome but not whole genome sequencing, a much higher SNV burden was found, including many recurrently mutated genes. However these data are difficult to interpret because of the differences in the mouse models used, the experimental design and methods of analysis, which did not include filtering of SNVs through exactly matched normal control DNA samples (see also Chang, H.-H. et al.^52^).

A surprising feature of our experiments, which evaluated the effects of 50 cGy high or low LET radiation, was the exceptionally high incidence of radiation-induced mammary cancers, comprising carcinomas and a range of sarcoma types including angiosarcomas. Other models of radiation-induced tumour development have not generally resulted in a high incidence of mammary cancers. This could be due to the effects of higher radiation dose (in the 2–5 Gy range) which is associated with earlier onset haematologic malignancies and lymphomas. Alternatively, it could be due to a combination of dose and genetic background, which could result in early death due to aggressive lymphoma or sarcoma development before the time when secondary carcinomas would arise, as the median tumour free survival was 267 and 327 days for mammary and lung tumours while it was only 130 days for lymphomas. This effect is further accentuated in gamma-IR (40 day latency in lymphoma versus 331 days in mammary), which has been more frequently used in historical studies. The present model therefore more closely mimics susceptibility to human secondary breast cancer development, which is known to be increased following radiation exposure and to be influenced by both germline TP53 and somatically acquired deficiency^20,53–55^.

High LET radiation appeared to be a more potent carcinogen when compared to the same dose of gamma rays, causing early onset tumours and increased tumour burden in the majority of tissue types. While it should be cautioned that in the absence of a dose-response evaluation one cannot ascertain the relative biological equivalence of the doses of gamma versus Fe-ion radiation used in these experiments, these findings nevertheless agree with prior work showing increased colon tumourigenicity induced by Fe ions compared to gamma radiation in the APC/Min mouse model^56^. Heavy-ion exposure was also associated with an increased proportion of carcinomas, and despite equal numbers of animals receiving the two radiation qualities, the incidence of the two most commonly observed tumours (mammary and lung) was significantly different for ^56^Fe-ions compared to gamma radiation. Furthermore, *Trp53* genotype impacts tumour histology, as mice hemizygous for p53 were more likely to develop sarcomas and spindle tumours, independent of radiation quality. In contrast, the incidences of squamous cell carcinomas and lymphomas were 10-fold and 5-fold higher, respectively, in *Trp53ΔP* versus Trp53+/− mice (Fig. 1c).

Our data on the patterns of genomic alterations in these different tumour types may help to rationalise these observations. On average, carcinomas exhibited higher numbers of complex chromosome aberrations, while sarcomas that arose in the same cohort of mice were genomically much more stable (Fig. 3c). Heavy ion exposure was strongly associated with an increased fraction of focal SVs, deletions and gene copy number changes relative to gamma exposure, particularly in mice carrying the *Trp53ΔP* allele. As a result, the *Trp53ΔP* genotype in combination with ^56^Fe-ion exposure resulted in the highest incidence of genomically unstable carcinomas. We conclude that heavy ion radiation exposure is, on average, more likely to generate the level of chromosomal instability required for transformation of the appropriate cell of origin for carcinomas.

Over 60% of mammary tumours, regardless of germline *Trp53* status, showed rearrangement and high-level amplification of the *Met* locus on chromosome 6. These tumours were predominantly induced by heavy ion radiation, which is compatible with the genome-wide observation of increased focal genomic events associated with exposure to ^56^Fe-ions. The propensity of heavy ion radiation to induce focal DNA damage, together with a specific feature of *Met* signalling in the mammary gland, may select for common tissue specific activation of this pathway.

Several examples were observed of tumours with complex patterns of whole chromosome or focal SVs, particularly in chromosome 1 and focal regions on chromosomes 6, 11, and 17. These features are similar to those generated by chromothripsis^23^ in human tumours. A strong interaction between germline *Trp53* status and radiation quality in determining the genomic changes in tumours is exemplified by analysis of chromosome 11, which harbours the *Trp53* gene. The simplest way to lose *Trp53* function completely in *Trp53+/−* mice is to lose the wild type allele, by chromosome non-disjunction, or by more focal events which can be induced by heavy ion radiation (Fig. 7). The complete loss of function in *Trp53ΔP* mice is less likely to occur due to the presence of two partially functional alleles on different chromosomes. Simple non-disjunction would not lead to loss of function, and we only saw loss of *Trp53ΔP* alleles in tumours induced by ^56^Fe-ions (Fig. 7), presumably due to the increased probability of focal DNA damage at the *Trp53* locus. These data emphasise the importance of interactions between inherited germline alleles and environmental exposures in determining tumour genome architecture.

Finally, our data shed light on a long-standing question regarding mechanisms of DNA damage induced by ionizing radiation. Double strand breaks can be generated by a direct interaction between radiation particles and DNA, or indirectly by generation of hydroxyl radicals, leading to formation of oxidised bases and DNA single or double strand breaks. Deconvolution of the mutational signatures in mammary tumours identified a significant contribution from several known human signatures, including signature 18 attributed to the generation of ROS^39,40^. Although the absolute number of point mutations attributable to this signature is relatively low, it was more prevalent in those tumours induced by gamma exposure compared to high energy ^56^Fe-ions (*P* < 0.0318, Fig. 2b). High energy radiation, on the other hand, is more likely to damage DNA directly causing complex focal genomic changes leading to a higher level of genetic instability and more aggressive tumours. While there are some parallels between this work on mouse models and sequence analysis of radiation-associated human cancers^4^, detailed comparisons will require a much more complete analysis of the genetic alterations in human cancers that are more definitively linked to radiation of different qualities and exposure levels.

## Methods

### Colony maintenance and breeding

The *Trp53ΔP* allele was initially maintained on a mixed 129/SvJ and C57BL/6J background as has been described previously^57^.We bred the *Trp53ΔP* allele from the mixed 129/Sv and C57BL/6 background onto the FVB/N background with greater than 10 crosses resulting in 99.9% FVB/N background for these mice. The *Trp53*^(*+/−*)^ allele has been previously bred onto the FVB background^35^. All mouse experiments received ethical approval by the University of California San Francisco Laboratory Animal Resource Centre. We have complied with all relevant ethical regulations for animal testing and research.

### Irradiation

To assess the effect of dose and compare HI to gamma radiation, we exposed mixed (129/C57) and pure (FVB/N) background mice to radiation. Mice were between 6 and 10 weeks of age. Mice exposed to HI radiation were subjected to 29 cGy, 50 cGy, 81 cGy or 1 Gy of 600 MeV/amu ^56^Fe-ions at a dose rate of 50 cGy/min delivered by a 20 × 20 cm beam. Gamma radiation consisted of 50 cGy or 1 Gy of ^137^Cs gamma rays at a dose rate of 25 cGy/min. Irradiation was performed at the Brookhaven national laboratory. Mice were shipped from UCSF to Brookhaven National Laboratories and rested for at least 3 day prior to whole-body irradiation. Following irradiation, mice were returned to UCSF for monitoring. Sham irradiated mice were flown to Brookhaven but were not irradiated.

### Tumour analysis and pathology

Mice were monitored for the development of tumours at UCSF by both visual evaluation and palpation for a minimum of 500 days, until the mouse body-condition score deteriorated, or a tumour exceeded 1 cm in diameter.

At the time of sacrifice radiosensitive tissues including the skin, mammary, thymus and spleen were harvested along with the tumour and the tail as a non-radiation sensitive control. A small section of each tissue was preserved for histology and the remainder was frozen for DNA, RNA or protein extraction. H&E slides were prepared from tumour samples and examined by a board-certified pathologist to determine histological subtypes. Tumours were categorised based on histology and disease site at time of necropsy. Mammary tumours include carcinomas, spindle cell tumours, sarcomas and angiosarcomas, the latter which was verified by immunohistochemistry (CD31+). To accurately represent the range of mammary tumours identified, whole exome sequencing analysis was performed on all mammary tumour types. Only carcinomas and sarcomas (angiosarcomas were excluded) were used for whole genome sequencing.

### DNA and RNA extraction

DNA was extracted from snap frozen tumour tissues using the Qiagen DNAeasy kit as per manufacturer’s instructions. Concentration and quality were determined by Nanodrop spectrophotometry and by PicoGreen (Invitrogen). RNA was extracted from snap frozen tissues using the Zymo Quick-RNA column purification kit. RNA concentration and quality were determined by Nanodrop and Agilent RNA Nano Bioanalyzer kit.

### Whole exome sequencing

Exonic DNA was captured using the Agilent whole exome capture kit (SureSelect Mouse All Exon). Captured material was indexed and sequenced on the Illumina platform at the Wellcome Trust Sanger Institute at an minimum depth of 100×. Raw 75 bp pair end sequencing reads were aligned with BWA-aln (v0.5.10)b^58^ to the GRCm38 mouse reference genome and were subsequently realigned around known InDels using GATK InDel re-aligner (v1.5-9) producing a single Binary Alignment Mapping (BAM) file for each sample.

### Whole genome sequencing

DNA was sheared to 450 bp fragment size using a Covaris S2. A single Illumina TruSeq v3 sequencing library was created for each strain according to manufacturer’s protocols. Each library was sequenced on a HiSeq 2000 over four lanes. Sequencing reads from each lane were aligned to the C57BL/6 J GRCm38 (mm10) mouse reference genome using BWA-MEM (0.7.13). For each library, aligned reads from each lane were merged using Picard Tools (v1.64) resulting in a single BAM file for each sample with a median coverage of 39×.

### SNV analysis

SNV calling for exomes was performed using MuTect (version 1.1.6)^59^. Tumour samples were called against two genotype and strain matched controls and filtered for quality. The following filters were used: minimum of 10 q20 or greater covering bases in both tumour and normal, minimum tumour alternate allele fraction of 0.2, minimum of 3 alternate alleles in tumour, no more than 3 alternate alleles in either normal. Variants were also filtered for known variation using the Sanger variant dataset (mgp.v3.snps.rsIDdbSNPv137, filtered to exclude wild mice and all variants with *q* less than 20), and a pooled set of variants and variable regions observed in all control samples (called by MuTect2 version 3.6)^59^.

Evaluating the result revealed the existence of a large number of likely germline variants in non-coding regions. To remove these variants, we selected all variants that were exactly replicated (same location, same alternate allele) in at least three samples without a consequential coding effect. In all cases these variants appeared to be approximately heterozygous, and no variant was consequential. As a result, we omitted all variants exactly replicated in three or greater samples without an effect on protein sequence, and all variants exactly replicated seven or more times without regard to context.

Initial analysis revealed the existence of a large number of variants with the motif GGTGN. These sites were overwhelmingly associated with T > G transversions resulting in a GGGG homopolymer. Sanger sequencing showed that the majority of these variants were false positives. We imposed an additional, substantially more stringent, set of filters for these sites: minimum of 50 q20 or greater covering bases in both tumour and normal, minimum tumour alternate allele fraction of 0.2, minimum of 15 alternate alleles in tumour, normal alternate allele fraction of no greater than 0.05. This dramatically reduced the influence of this artifact. After this filtration, we took the intersection of the pass-filter variants between both controls as the keeper set for each tumour.

For WGS. somatic variants were detected using CaVEMan, an expectation maximisation–based somatic substitution detection algorithm^60^. Detected somatic variants were then filtered using an array of quality filters and common mouse genome variants were excluded. Tumours were called against paired tail DNA and filtered for the following; greater than or equal to 10 q20 or more bases, alternate allele fraction greater than 0.1 in tumour, and alternate allele fraction less than 0.1 in normal. In addition, we removed all variants occurring in simple repeat regions (simpleRepeat.txt, downloaded from hgdownload.cse.ucsc.edu/goldenpath/mm10/database/)^61^.

### Indel analysis

Indel calling in exome samples was performed using pindel (version 0.2.4w;^62^). Samples were called against two normal with the following options: -l false –x 1 –M 4. Results were then filtered for germline events, recurrence, and simple repeats. Germline filtration was accomplished by identifying all sites found in controls in this cohort and flagging any tumour indel that overlapped (within 2 bases) these sites. We also repeated this strategy for germline events found in the Sanger list of indel events (mgp.v3.indels.rsIDdbSNPv137, filtered for minimum quality 20 and no variants present only in wild derived strains).

We noticed a large degree of exact recurrence of indels within the tumour cohort, even following germline filtration. After manual examination, we determined the majority of these variants were likely to be germline or the result of recurrent sequencing/calling artifacts. To remove these variants, we removed all non-consequential indels that were exactly replicated (same location and alternate allele) in 3 or more samples. We also flagged regions (50 bp windows) containing three or more indels of any type. Manual evaluation of these regions revealed them to be primarily artefactual, with a small number of potentially biologically meaningful sites. As the number of artefactual variants substantially outnumbered the number of biologically meaningful sites, we omitted these regions for the calculation of indel rates but included them for the analysis of functional impact on cancer associated genes. We also removed all exome indels in simple repeat regions.

Indels were called in whole-genome samples by comparing tumour to paired control tail DNA using a modified version of the pindel programme^62^. Resulting lesions were then filtered for exact and regional recurrence (more than three exact replicates or indels within 50 bp across the tumour cohort), and presence within simple repeat regions. Indel rates were defined similar to SNVs.  

### Mutation signature analysis

The mutational catalogues from the 21 WGS samples were analyzed for mutational signatures. Signature extraction was performed using SigProfiler (conducted using methods based on nonnegative matrix factorisation (NMF) as described in Alexandrov et al. (refs. ^1,36,37^)). Analyses were carried out separately for single base substitutions (SBS signatures) and indels (ID signatures). We first performed de novo mutation signature extraction using NMF-based method as described in SigProfiler.

For SBS signatures, extracted signatures were then compared to the set of mutational signatures deciphered from the COSMIC database as previously described (refs. ^1,36,37^). The algorithm identifies the optimal combination of known human signatures that explains the observed mutation patterns (highest cosine similarity). All signatures extracted across all samples were able to be explained by a combination of signatures from the known human signatures (cosine similarity > 0.75) and thus we did not identify any signatures that would be considered novel. We performed hierarchical clustering of the samples based on the relative contribution of identified mutation signatures using both results from the de novo extraction and after signature deconvolution.

For INDELs, given the limited understanding for these signatures, particularly in mouse, as well as the fact that there were very few mutations, we chose to perform downstream analysis using only the de novo extracted signatures.

Code for the original SigProfiler software is available: https://www.mathworks.com/matlabcentral/fileexchange/38724-sigprofiler.

For further details of this algorithm and it’s most up to date implementation we refer the reader to ref. ^1^ and the updated software webpage: https://pypi.org/project/sigprofiler/.

### CNV segmentation and analysis

CNV segmentation in exomes was performed using CNVkit^63^ following the default workflow with target regions defined from the UCSC refgene flatfile gene locations padded by +/−500 basepairs.

To remove spuriously aberrant regions we assessed control copy-number profiles for aneuploidies. Any region found to be aberrant in two or more controls was omitted from the tumour samples.

CNV segmentation in whole-genome samples was performed using BICseq (version 1.1.2;^64^) with the following options: --paired --I = 400,50 --bin_size = 250. Three samples showed consistently patterned aneuploidies that appeared to be artefactual and were omitted (MD5215a, MD5212a, MD5210a).

Amplification was defined as a copy-number log2 ratio over 0.15, and deletion as log2 ratio less than −0.15. A whole-chromosome amplification was defined as greater than 50% of the chromosome covered by copy-number bins, and greater than 40% of the chromosome called as amplified. Whole-chromosome deletions were defined with similar proportions. Mixed chromosomes were chromosomes that fit the definition of amplification or deletion, but with 5% or more of the chromosomes total length aneuploid in the opposite direction. This definition captured the majority of the visibly mixed chromosomes without including any discernable false positives.

Significantly recurrent whole-chromosome aneuploidies were identified by first permuting chromosome state per sample 1000 times, counting the maximum observed quantity of aneuploidies per chromosome in the randomised data, and identifying the threshold that was greater than 95% of observed maximum aneuploidy counts in the permuted data. This was done for any aneuploidy, amplifications only, and deletions only.

Focal amplifications and deletions were identified through the cghMCR package^65^ with amplification and deletion thresholds increased to +/−0.25 to focus on high-level aneuploidies. Significance threshold was defined by 1000 permutations as above.

#### Rearrangement analysis

Rearrangements were called using both lumpy^66^ and breakdancer^67^ but only breakdancer calls were used due to extremely high agreement between the intrachromosomal events from both callers, and the high level of spurious interchromosomal rearrangements identified by lumpy. These spurious calls appeared to corroborate recent findings showing that lumpy performed poorly when calling non-C57B6 samples against the C57BN6 genome^68^. Breakdancer was used with the following arguments: -r 10 –q 30.

Rearrangements were filtered by depth and region. Depth filters included the following: less than 2 control reads, and greater than or equal to 10 tumour reads. Regional filters included simple repeats and mapability. Mapability was defined as uniquely mapping from the crgMapabilityAlign50mer.txt file for the mm9 genome (downloaded from hgdownload.cse.ucsc.edu/goldenpath/mm9/database) remapped to mm10 with liftOver^61^. Any rearrangement with either endpoint within a simple repeat or non-uniquely mapping region was omitted.

Focal rearrangement hotspots were identified by establishing the intermutation distance (the distance between any given rearrangement and its nearest neighbour) in 1 Mb windows sliding by 100 kb intervals. Enriched sites were defined as those windows with intermutation distance greater than five standard deviations greater than the genome-wide mean, with at least six unique lesions, in at least three different samples.

#### Statistical analysis and plotting

Statistical analysis was performed in R (version 3.3.3)^69^. Two-group analyses were performed using the non-parameteric Mann-Whitney U test. Fisher’s test was used to assess categorical data. The *glm* function was used to model the relationship between whether or not a chromosome was aneuploid and genotype/radiation exposure. Survival analysis was performed using Cox proportional hazards modelling and significance evaluated by the log-rank test. Plots were generated using the *ggplot2* package^70^.

### Reporting summary

Further information on research design is available in the Nature Research Reporting Summary linked to this article.

## Supplementary information

Supplementary Information

Reporting Summary

Description of Additional Supplementary Files

Supplementary Data 1

Supplementary Data 2

Supplementary Data 3

Supplementary Data 4

## Data Availability

The whole-exome sequencing data have been deposited in the ENA database under the accession code ERP001454 [https://www.ebi.ac.uk/ena/data/view/PRJEB3044]. The whole-genome sequencing data have been deposited in the ENA database under the accession code ERP001454 [https://www.ebi.ac.uk/ena/data/view/PRJEB13771]. All the other data supporting the findings of this study are available within the article and its supplementary information files and from the corresponding author upon request.

## Code availability

All software utilised for this paper are publically available and are as indicated in the Methods section. Any custom code or scripts used in this paper are available upon request.
