# Supplementary material for: Mutational signatures in tumours induced by high and low energy radiation in Trp53 deficient mice
Source: Nat Commun. 2020 Jan 20;11:394. doi: 10.1038/s41467-019-14261-4 (PMC6971050; doi:10.1038/s41467-019-14261-4)
Supplement: Supplementary file 1 — Supplementary Information [file 41467_2019_14261_MOESM1_ESM.pdf]

Supplementary Information

Mutational signatures in tumours induced by high and low energy radiation in Trp53 deficient mice.

Li et al.

Supplementary Figure 1

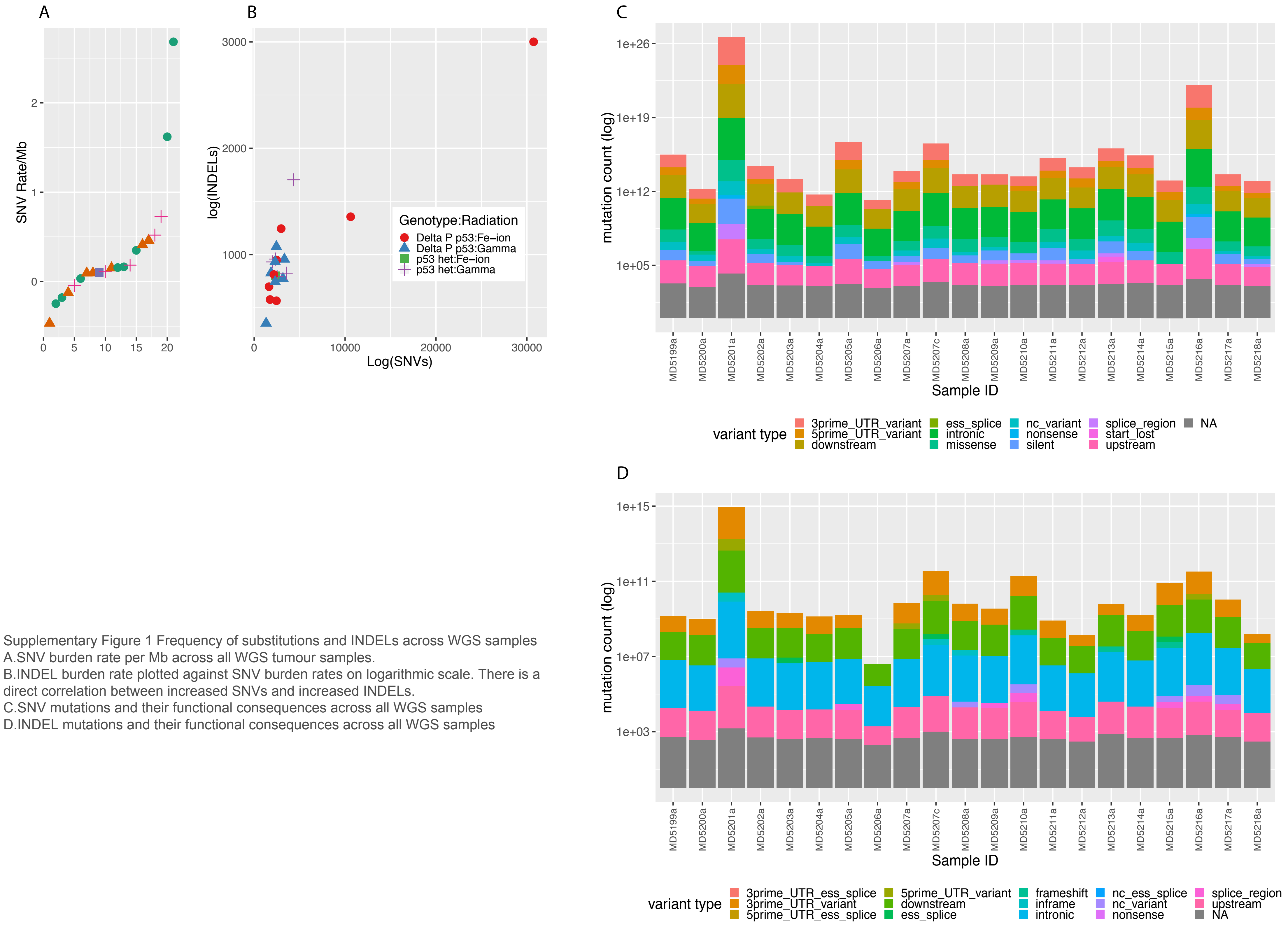

Supplementary Figure 1 Frequency of substitutions and INDELs across WGS samples
A.SNV burden rate per Mb across all WGS tumour samples.
B.INDEL burden rate plotted against SNV burden rates on logarithmic scale. There is a direct correlation between increased SNVs and increased INDELs.
C.SNV mutations and their functional consequences across all WGS samples
D.INDEL mutations and their functional consequences across all WGS samples

Supplementary Figure 2

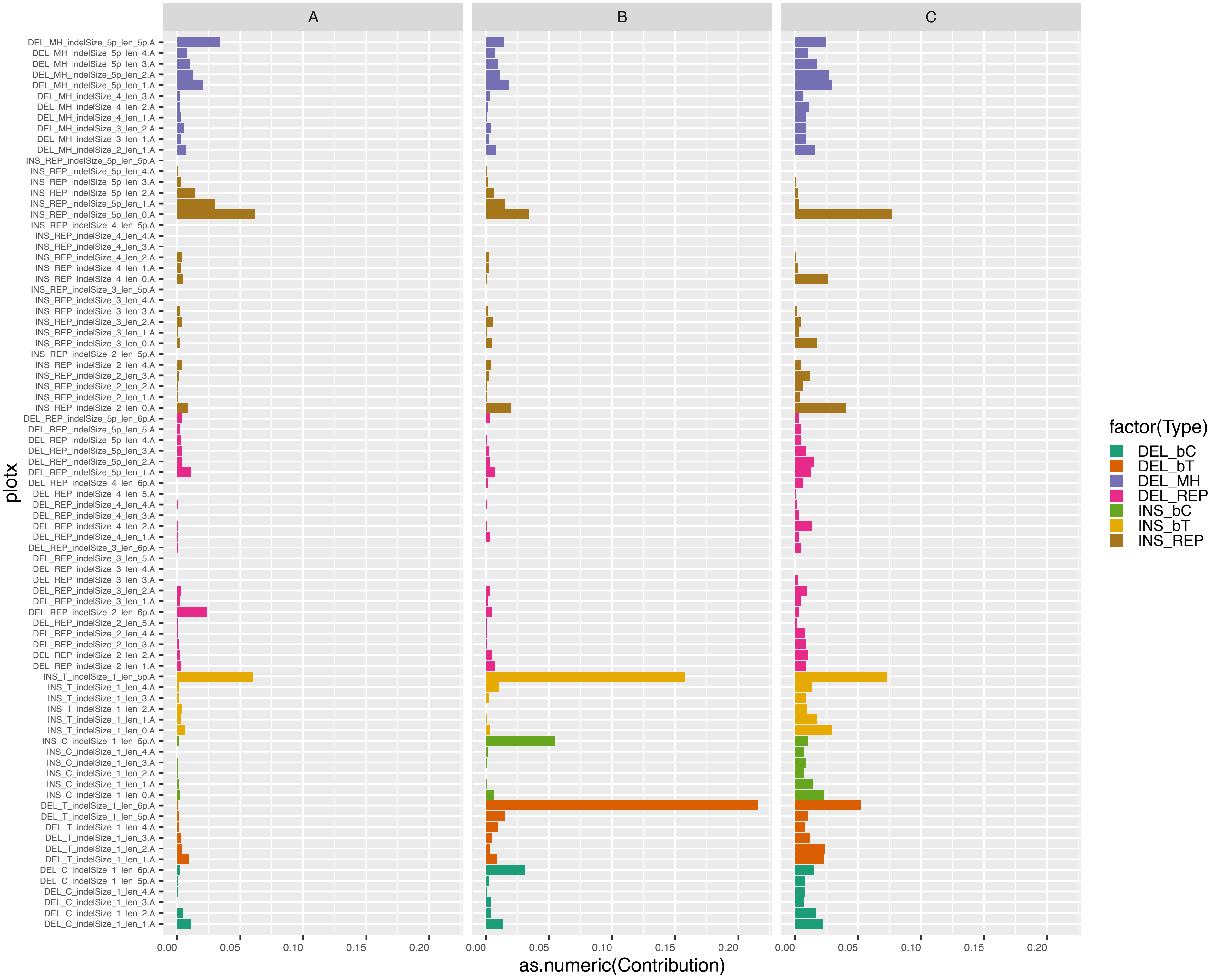

Supplementary Figure 2 Patterns of INDELs in samples from whole genome sequencing
Relative contribution of each insertion/deletion mutation type in the three INDEL signatures identified from the mammary samples. Detailed figure expansion of each type of INDEL variant group identified also in the condensed main Figure 2B. “Del_b” = 1bp deletion, “Ins_b” = 1bp insertion, where “C” or “T” describes whether cytosine or thymidine was deleted at that position, “MH” = microhomology domain and “REP” = >1bp deletion or insertion at repeats.

Supplementary Figure 3

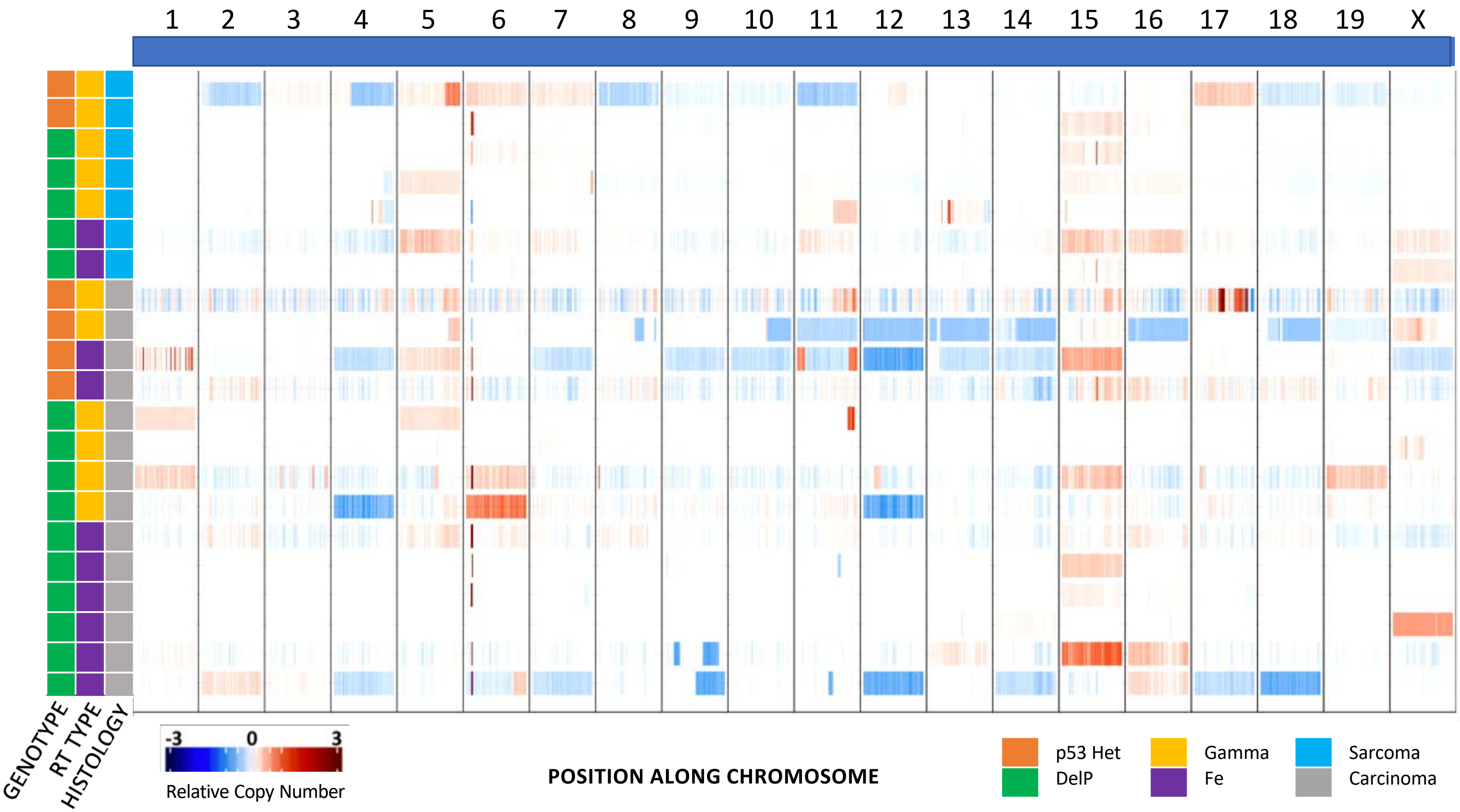

Supplementary Figure 3 Copy number variations across all whole-genome sequenced samples. CNVs across all whole genome sequenced samples. Tumour samples are sorted by tissue site and subsequently by pathology, radiation quality and genotype status of Trp53. Color code on the left indicates germline Trp53 genotype, radiation quality, and tumour histology (sarcoma or carcinoma). Relative copy number states across each chromosome are indicated by a corresponding key as shown at the lower left. Some chromosomes show predilection for copy number gains (eg. Chromosomes 5 and 15) while other regions show more deletions (Chromosome 4 or 12) or focal amplifications (e.g. proximal Chromosome 6 corresponding to Met)

Supplementary Figure 4

A

Met containing region selected by amplification status

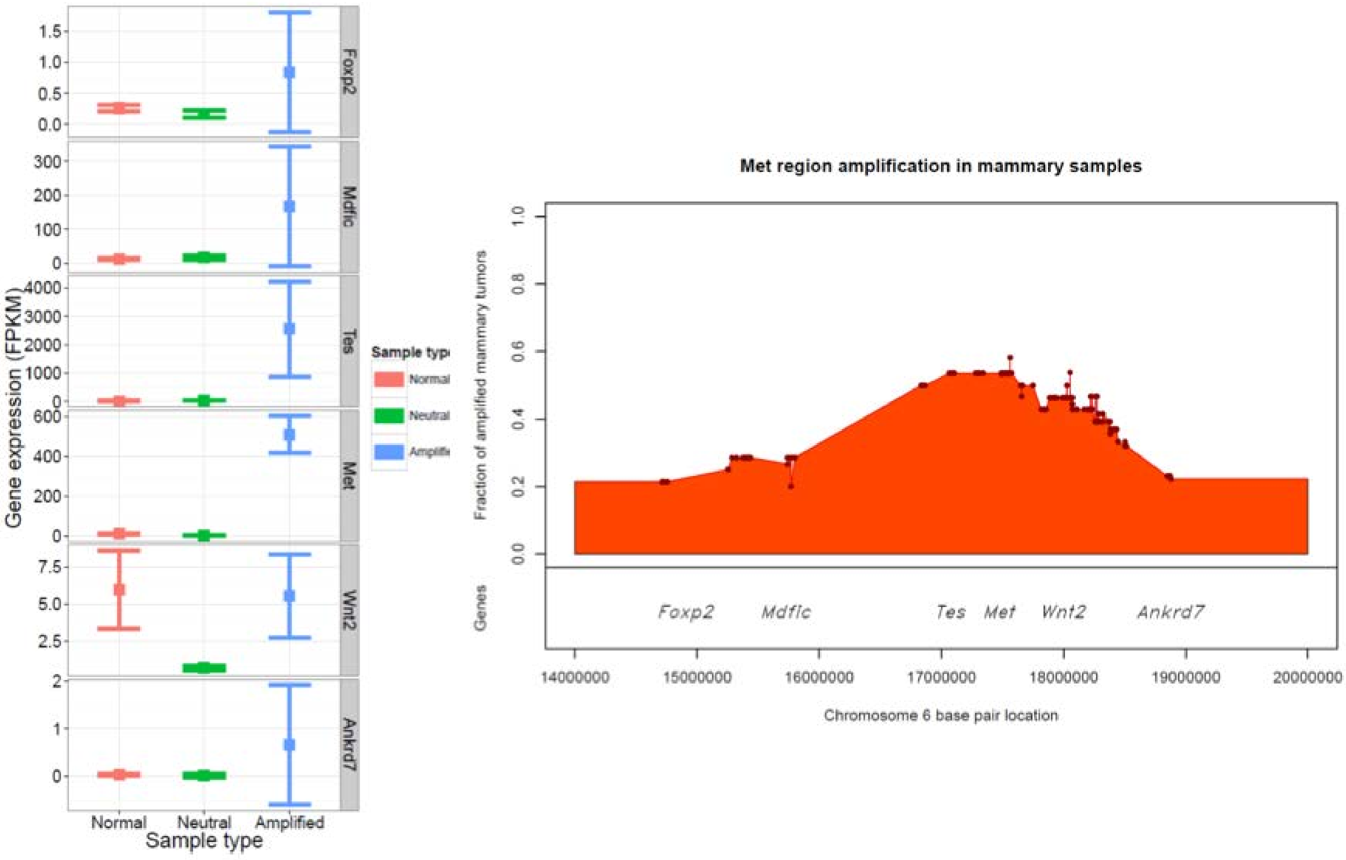

B

Mmp13 containing region selected by amplification status

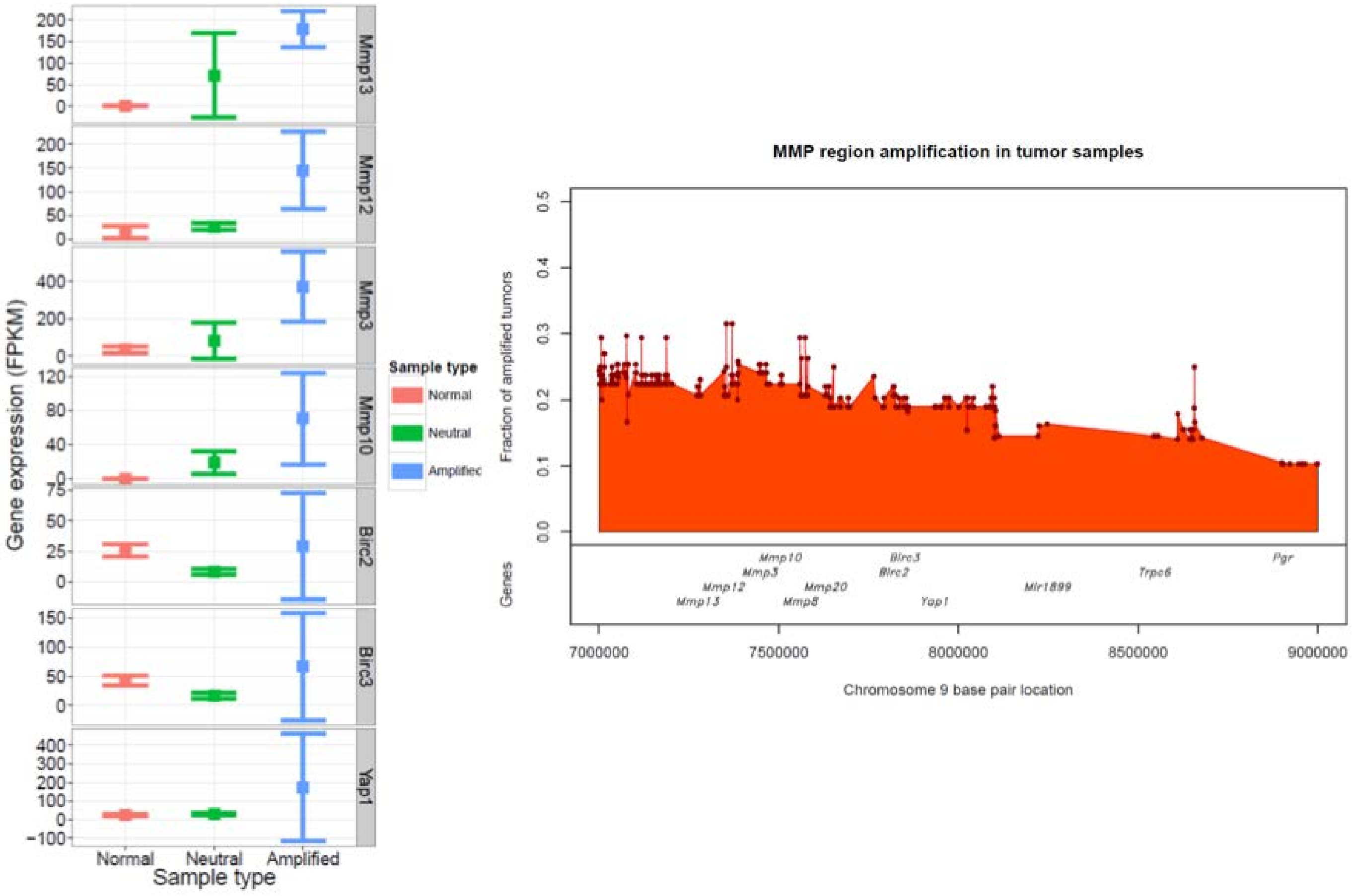

Supplementary Figure 4 Copy number gains of Met and Mmp13 genes are supported by expression analysis of these genes
A.Higher expression of Met on chromosome 6 is strongly associated with copy number status. Examination of the Met locus showing there is a corresponding increase in the density of samples with focal amplifications with increased density of structural variants.
B.Higher expression of Mmp13 on proximal chromosome 9 is associated with increased copy number status. Examination of the Mmp13 locus showing there is a corresponding increase in expression of several genes in this cluster including Mmp13, Mmp12, Mmp3, and Mmp10. Some other genes within the same region (Birc2, Birc3 and Yap1) show only marginal changes in expression levels.

Supplementary Figure 5

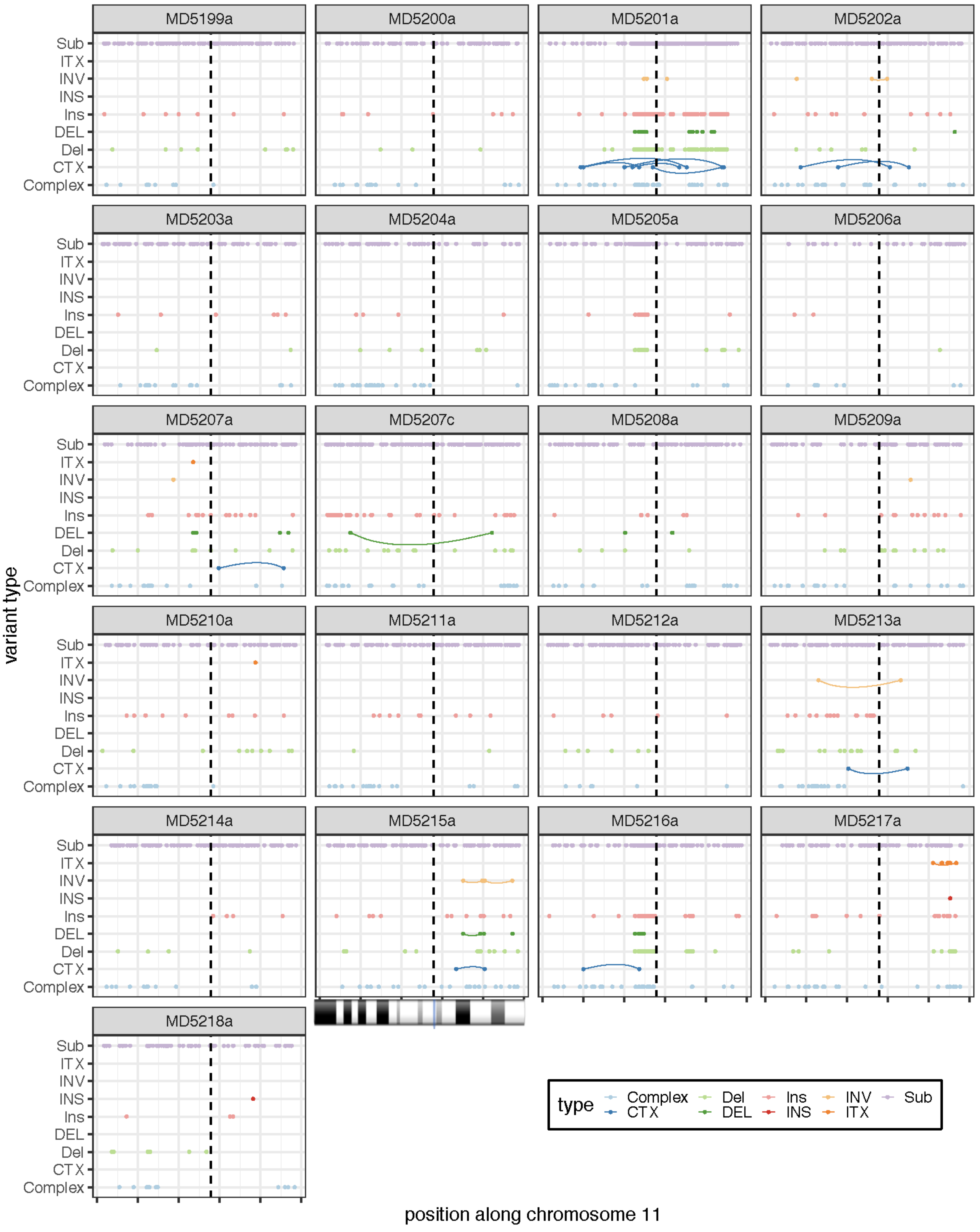

Supplementary Figure 5 Focal enrichment of SVs across chromosome 11
Plot of all mutations by variant type on chromosome 11 across whole-genome sequenced tumours, some with evidence of co-localization of breakpoints and/or variants. X-axis denotes position of chromosome 11. SNVs and SVs show preferential enrichment overlap particularly towards the distal half of the chromosome 11 corresponding to the region overlapping the locus encoding Trp53. Sub = Substitutions, ITX = Intrachromosomal translocation, INV = Inversion rearrangements, INS = Insertion rearrangements, DEL = Deletion rearrangements, CTX = Interchromosomal translocation, Ins = Small Insertions, Del = Small deletions, Complex = Complex INDELS. Dotted line denotes the location of Trp53 on chromosome 11.

Supplementary Figure 6

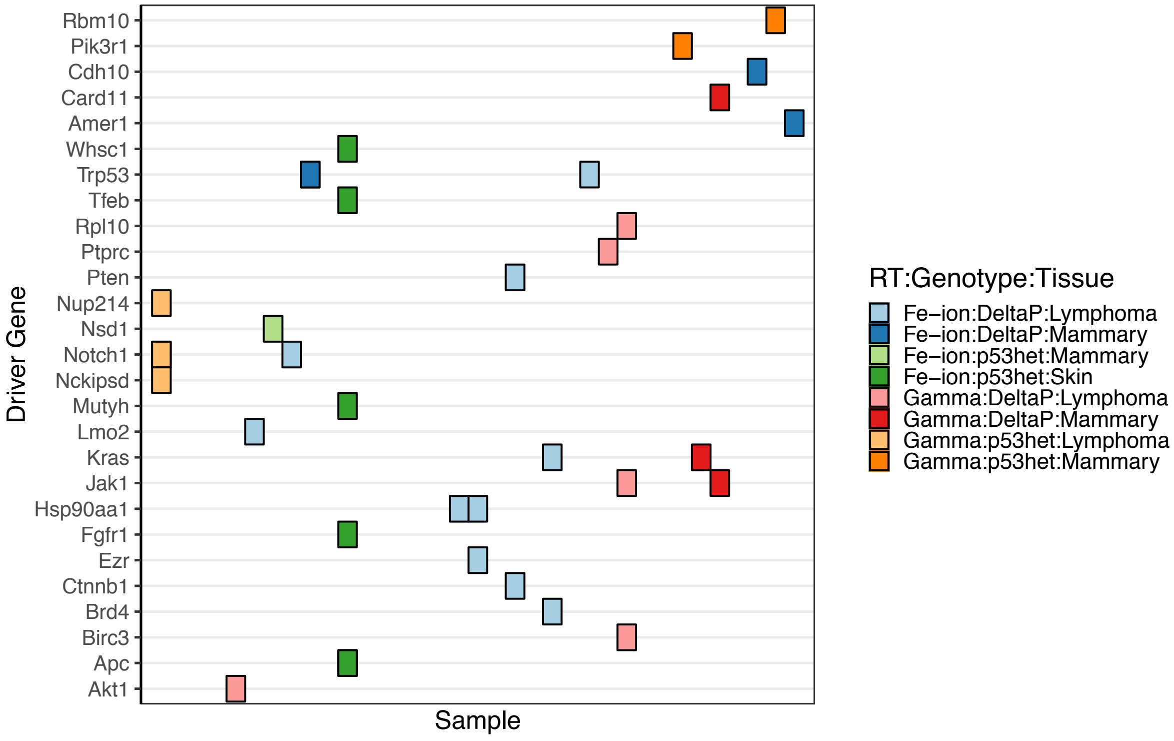

Supplementary Figure 6 Somatic mutations in driver genes in radiation-induced malignancies
Driver mutations across all sequenced samples. Each unique sample is one column across the x-axis and colored by radiation quality, Trp53 genotype and cancer tissue site.

Supplementary Table 1 Whole genome sequenced tumor samples

WGS ID	Mouse number	Genotype	Radiation	Radiation dose	Pathology	Sample type
MD5199a	AFVB 207	Delta P p53	Gamma	0.5 Gy	Carc	Tumor
MD5200a	FVB 1142	Delta P p53	Fe-ion	0.5 Gy	sarcoma	Tumor
MD5201a	FVB 1168	Delta P p53	Fe-ion	0.5 Gy	Carc	Tumor
MD5202a	FVB 1174	Delta P p53	Fe-ion	0.5 Gy	Carc	Tumor
MD5203a	FVB 1287	p53 het	Fe-ion	0.5 Gy	Carc	Tumor
MD5204a	FVB 1420	Delta P p53	Gamma	0.5 Gy	sarcoma	Tumor
MD5205a	FVB 1454	Delta P p53	Gamma	0.5 Gy	sarcoma	Tumor
MD5206a	FVB 1463	Delta P p53	Gamma	0.5 Gy	Carc	Tumor
MD5207a	FVB 1648	p53 het	Gamma	0.5 Gy	sarcoma	Tumor
MD5207c	FVB 1648	p53 het	Gamma	0.5 Gy	Carc	Tumor
MD5208a	FVB 1655	p53 het	Gamma	0.5 Gy	Carc	Tumor
MD5209a	FVB 1951	Delta P p53	Fe-ion	0.5 Gy	sarcoma	Tumor
MD5210a	FVB 1956	Delta P p53	Gamma	0.5 Gy	Carc	Tumor
MD5211a	FVB 1957	Delta P p53	Gamma	0.5 Gy	sarcoma	Tumor
MD5212a	FVB 1976	Delta P p53	Fe-ion	0.5 Gy	Carc	Tumor
MD5213a	FVB 1998	Delta P p53	Fe-ion	0.5 Gy	Carc	Tumor
MD5214a	FVB 2023	p53 het	Gamma	0.5 Gy	sarcoma	Tumor
MD5215a	FVB 2046	p53 het	Gamma	0.5 Gy	Carc	Tumor
MD5216a	FVB 2059	Delta P p53	Fe-ion	0.5 Gy	Carc	Tumor
MD5217a	FVB 2066	Delta P p53	Gamma	0.5 Gy	Carc	Tumor
MD5218a	FVB 2078	Delta P p53	Fe-ion	0.5 Gy	Carc	Tumor

List of tumour and corresponding normal tail samples collected for all tumours that underwent whole genome sequencing. All samples were mammary tumours. Also included is the radiation type, Trp53 genotype and histology.
