# Supplementary material for: Mutational signatures in tumours induced by high and low energy radiation in Trp53 deficient mice
Source: Nat Commun. 2020 Jan 20;11:394. doi: 10.1038/s41467-019-14261-4 (PMC6971050; doi:10.1038/s41467-019-14261-4)
Supplement: Supplementary file 2 — Reporting Summary [file 41467_2019_14261_MOESM2_ESM.pdf]

nature research

Corresponding author(s): Allan Balmain

Last updated by author(s): 11/29/2019

Reporting Summary

Nature Research wishes to improve the reproducibility of the work that we publish. This form provides structure for consistency and transparency in reporting. For further information on Nature Research policies, see Authors & Referees and the Editorial Policy Checklist .

Statistics

For all statistical analyses, confirm that the following items are present in the figure legend, table legend, main text, or Methods section.

n/a	Confirmed	
☐	☒	The exact sample size (*n*) for each experimental group/condition, given as a discrete number and unit of measurement
☐	☒	A statement on whether measurements were taken from distinct samples or whether the same sample was measured repeatedly
☐	☒	The statistical test(s) used AND whether they are one- or two-sided<br>*Only common tests should be described solely by name; describe more complex techniques in the Methods section.*
☐	☒	A description of all covariates tested
☐	☒	A description of any assumptions or corrections, such as tests of normality and adjustment for multiple comparisons
☐	☒	A full description of the statistical parameters including central tendency (e.g. means) or other basic estimates (e.g. regression coefficient) AND variation (e.g. standard deviation) or associated estimates of uncertainty (e.g. confidence intervals)
☐	☒	For null hypothesis testing, the test statistic (e.g. *F*, *t*, *r*) with confidence intervals, effect sizes, degrees of freedom and *P* value noted<br>*Give P values as exact values whenever suitable.*
☒	☐	For Bayesian analysis, information on the choice of priors and Markov chain Monte Carlo settings
☒	☐	For hierarchical and complex designs, identification of the appropriate level for tests and full reporting of outcomes
☒	☐	Estimates of effect sizes (e.g. Cohen's *d*, Pearson's *r*), indicating how they were calculated

Our web collection on statistics for biologists contains articles on many of the points above.

Software and code

Policy information about availability of computer code

Data collection	All data were collected either by direct observation (phenotypes such as date of tumor development, tumor pathology) or based on sequencing analysis. See supplemental methods for complete details on sequencing data analysis.
Data analysis	All software used are open source and available as noted in the supplemental methods. Custom codes in UNIX, PERL or R can be provided upon request.

For manuscripts utilizing custom algorithms or software that are central to the research but not yet described in published literature, software must be made available to editors/reviewers. We strongly encourage code deposition in a community repository (e.g. GitHub). See the Nature Research guidelines for submitting code & software for further information.

Data

Policy information about availability of data

All manuscripts must include a data availability statement. This statement should provide the following information, where applicable:

- Accession codes, unique identifiers, or web links for publicly available datasets
- A list of figures that have associated raw data
- A description of any restrictions on data availability

Raw genomic sequencing data will be deposited into the NIH open access repository GEO upon acceptance of the manuscript.

Field-specific reporting

Please select the one below that is the best fit for your research. If you are not sure, read the appropriate sections before making your selection.

☒ Life sciences ☐ Behavioural & social sciences ☐ Ecological, evolutionary & environmental sciences

For a reference copy of the document with all sections, see nature.com/documents/nr-reporting-summary-flat.pdf

Life sciences study design

All studies must disclose on these points even when the disclosure is negative.

Sample size	Sample size was estimated based on previous sequencing experience in the lab of tumors from similar mouse models exposed
Data exclusions	No data was excluded
Replication	Whole genome sequencing was initially used and findings were thereafter replicated and confirmed in whole exome sequencing data and genotyping assays for confirmed of identified variants and variant classes
Randomization	Not applicable
Blinding	Not applicable, although analysis

Reporting for specific materials, systems and methods

We require information from authors about some types of materials, experimental systems and methods used in many studies. Here, indicate whether each material, system or method listed is relevant to your study. If you are not sure if a list item applies to your research, read the appropriate section before selecting a response.

Materials & experimental systems

n/a	Involved in the study	
☒	☐	Antibodies
☒	☐	Eukaryotic cell lines
☒	☐	Palaeontology
☐	☒	Animals and other organisms
☒	☐	Human research participants
☒	☐	Clinical data

Methods

n/a	Involved in the study	
☒	☐	ChIP-seq
☒	☐	Flow cytometry
☒	☐	MRI-based neuroimaging

Animals and other organisms

Policy information about studies involving animals; ARRIVE guidelines recommended for reporting animal research

Laboratory animals	Mus musculus, FVB cross with 129 mixed genetic background with at least 10 generation backcross to FVB, both male and female animals were included (see supplemental tables) and animals were irradiated at 60-80 days of age and followed until development of tumor or animals became moribund
Wild animals	NA
Field-collected samples	NA
Ethics oversight	All experiments were approved by the UCSF IACUC

Note that full information on the approval of the study protocol must also be provided in the manuscript.
