# Supplementary material for: Mutational signatures in tumours induced by high and low energy radiation in Trp53 deficient mice
Source: Nat Commun. 2020 Jan 20;11:394. doi: 10.1038/s41467-019-14261-4 (PMC6971050; doi:10.1038/s41467-019-14261-4)
Supplement: Supplementary file 3 — Description of Additional Supplementary Files [file 41467_2019_14261_MOESM3_ESM.pdf]

Description of Additional Supplementary Files

File Name: Supplementary Data 1
Description: List of study animals, genotype, sex, radiation treatment, overall survival in days and presence of tumour in the animal upon sacrifice.

File Name: Supplementary Data 2
Description: List of tumour and corresponding normal tail samples collected for all tumours that underwent exome genome sequencing. All samples were mammary tumours. Also included is the radiation type, Trp53 genotype and histology.

File Name: Supplementary Data 3
Description: List of candidate loss of function and/or non-synonymous SNVs observed in tumour samples identified by sequencing.

File Name: Supplementary Data 4
Description: List of candidate loss of function and/or non-synonymous INDELS observed in tumour samples identified by sequencing.
